# How do the Fat–Dachsous and core planar polarity pathways act together and independently to coordinate polarized cell behaviours?

**DOI:** 10.1098/rsob.200356

**Published:** 2021-02-10

**Authors:** Helen Strutt, David Strutt

**Affiliations:** Department of Biomedical Science, University of Sheffield, Western Bank, Sheffield S10 2TN, UK

**Keywords:** planar polarity, planar cell polarity, PCP, Frizzled, Fat, Dachsous

## Abstract

Planar polarity describes the coordinated polarization of cells within the plane of a tissue. This is controlled by two main pathways in *Drosophila*: the Frizzled-dependent core planar polarity pathway and the Fat–Dachsous pathway. Components of both of these pathways become asymmetrically localized within cells in response to long-range upstream cues, and form intercellular complexes that link polarity between neighbouring cells. This review examines if and when the two pathways are coupled, focusing on the *Drosophila* wing, eye and abdomen. There is strong evidence that the pathways are molecularly coupled in tissues that express a specific isoform of the core protein Prickle, namely Spiny-legs. However, in other contexts, the linkages between the pathways are indirect. We discuss how the two pathways act together and independently to mediate a diverse range of effects on polarization of cell structures and behaviours.

## Graphical abstract

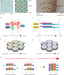

## 1. Introduction

Most epithelial tissues must be polarized in the plane of the tissue axis, to allow not only the formation of polarized subcellular structures, but also to direct the reorganization of cells in a coordinated, polarized fashion. This coordinated polarization is collectively known as planar polarity (also known as planar cell polarity or PCP) [1–3]. It can be visualized in structures such as body hairs, feathers and scales, and also microscopic features such as motile cilia, that all point in the same direction. In addition, planar polarity is evident in convergence and extension movements, where cells converge on one axis and elongate on the other.

**Figure 1.**
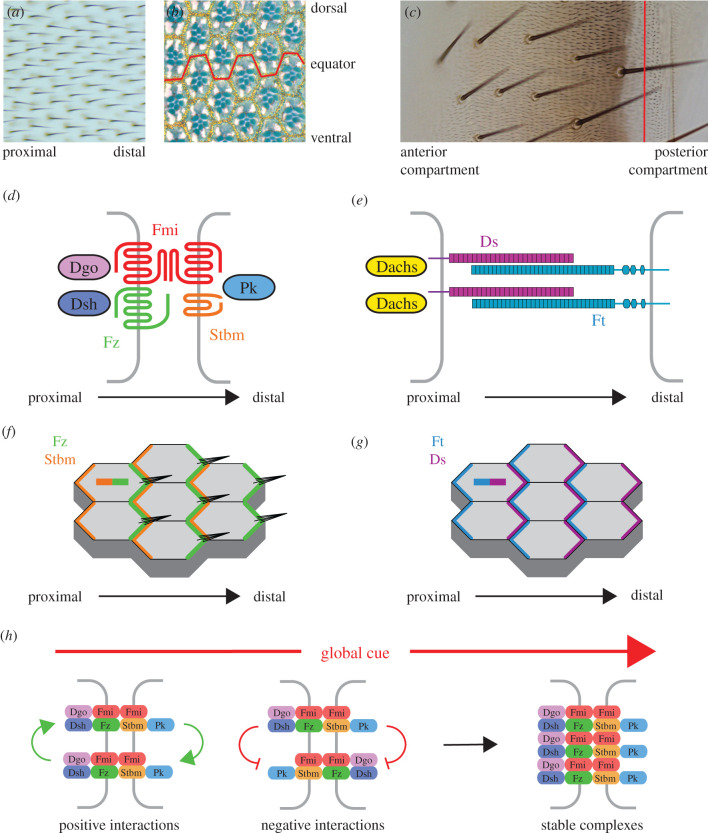
Planar polarity in *Drosophila*. (*a*) Image of the dorsal surface of an adult wing. Each cell produces a single trichome that points distally. (*b*) Image showing a section through an adult eye. Each ommatidium in the eye contains eight photoreceptor cells (stained blue) that are organized in a trapezoid pattern. Ommatidia on either side of the dorsoventral midline (equator, red) have opposite orientation. (*c*) Image of a single segment of the dorsal surface of an adult abdomen. The abdomen consists of eight segments, each of which is divided into anterior and posterior compartments (approximate position of compartment boundary indicated in red). It is a cuticular structure in which bristles and hairs point posteriorly. (*d*) Localization of the core planar polarity pathway components in the wing imaginal disc and pupal wing. Fz, Dsh and Dgo localize to distal cell ends, while Stbm and Pk localize to proximal cell ends. Fmi localizes proximally and distally, where it binds homophilically and mediates intercellular communication. (*e*) Localization of Ft–Ds pathway components in the wing imaginal disc. Ds and Dachs localize to distal cell ends, while Ft localizes proximally. Heterophilic binding between Ft and Ds is modulated by phosphorylation on their extracellular domains by the kinase Fj. (*f*) Asymmetric localization in a group of cells is shown by distal Fz (green) and proximal Stbm (orange). Core proteins localize to the apical adherens junction zone and promote distal localization of the trichome. The green/orange bar in the top left cell illustrates distally localized Fz and proximally localized Stbm (also in figures 2–5). (*g*) Asymmetric localization of distal Ds (purple) and proximal Ft (blue) in a group of cells. The purple/blue bar in the top left cell illustrates distally localized Ds and proximally localized Ft (also in figures 2–5). (*h*) Model for self-organization of the core proteins by feedback interactions. An initial bias in core protein activity is generated by a global cue. This bias is amplified by positive interactions, where complexes of the same orientation are stabilized (left), and negative interactions, where complexes of the opposite orientation are destabilized (middle). This leads to sorting of complexes into a uniform orientation (right).

Mechanisms underlying planar polarity are most well-characterized in the fruit fly *Drosophila melanogaster*, where all adult cuticular tissues exhibit planar polarized structures. These include hairs and bristles on the wing, abdomen, legs and notum and ommatidia in the eye (figure 1*a–c*). Two major pathways have been identified that control planar polarity in *Drosophila*: the ‘core’ planar polarity pathway and the Fat–Dachsous (Ft–Ds) pathway (figure 1*d*,*e*). However, other pathways exist that regulate, for example, egg elongation and planar polarized cell rearrangements during germ band extension in the *Drosophila* embryo, and these have been reviewed elsewhere [4–8].

Some reports have suggested that the Ft–Ds pathway acts upstream of the core, while others have argued that they can act independently. This review will discuss how Ft–Ds and the core pathways affect cell behaviours in three well-studied tissues—the *Drosophila* wing, eye and abdomen. We will examine the evidence for coupling between the two pathways, and discuss both direct and indirect mechanisms that act depending on tissue type and developmental stage. We will then discuss how this might be relevant in other invertebrate and vertebrate systems.

## 2. The core planar polarity pathway in *Drosophila*

The core pathway comprises six distinct proteins that form asymmetrically localized intercellular complexes. We will first describe the localization and function of the core proteins in the pupal wing, and then go on to describe their roles in the eye and abdomen.

### 2.1. The core pathway in the *Drosophila* wing

**Figure 2.**
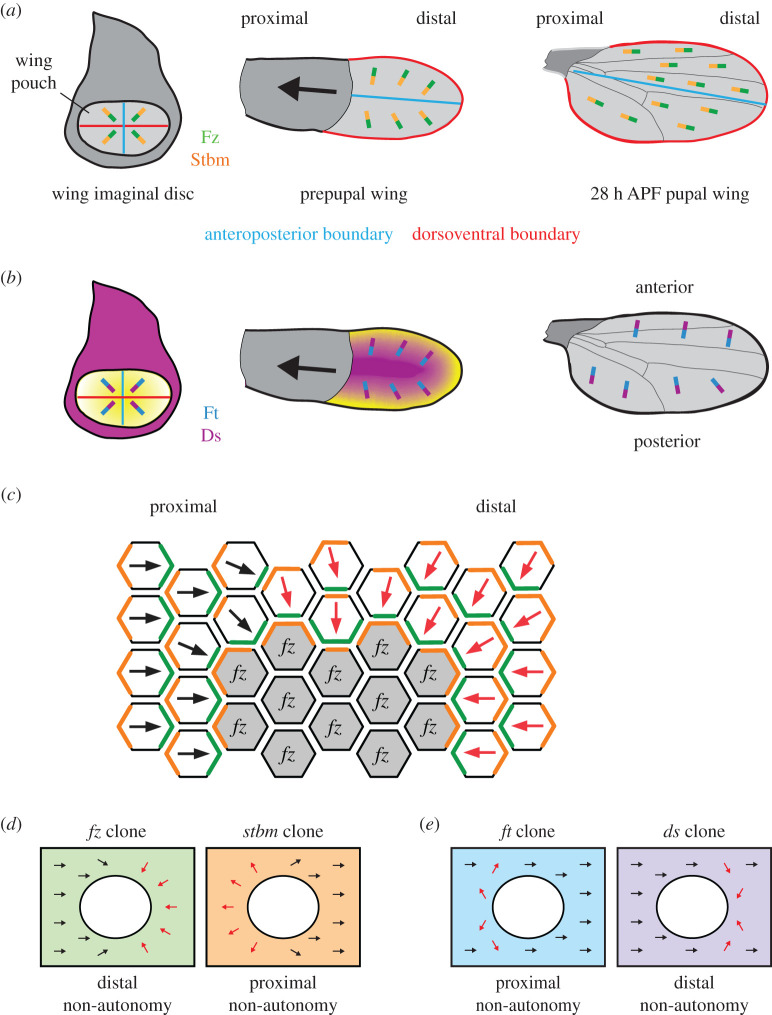
Planar polarity in the *Drosophila* wing. (*a*) Core protein localization in the wing disc (left), prepupal wing (middle) and pupal wing at 28 h after puparium formation (APF) (right). The anteroposterior boundary (blue) and dorsoventral boundary (red) are shown. The wing imaginal disc contains a central pouch (light grey) that forms the pupal wing, where the centre of the wing pouch is distal, and the outer circumference is proximal. The remainder of the wing disc (dark grey) forms the wing hinge and notum. The epithelium of the imaginal disc everts and extends, before folding over to form a double-layered epithelium in the pupal wing. Core protein localization in each cell is indicated by distal Fz (green bar) and proximal Stbm (orange bar). In the wing imaginal disc and prepupal wing the core proteins are localized radially. Hinge contraction causes tissue flows, leading to cell rearrangements and a redistribution of the core proteins, so that they align along the proximodistal axis of the pupal wing at 28 h APF. (*b*) Ft–Ds pathway expression and localization in wing disc (left), prepupal wing (middle) and 28 h APF pupal wing (right). In the wing imaginal disc, Ds is highly expressed in the hinge region (purple), while Fj is expressed in a gradient in the wing pouch, high distally (yellow). Ds is localized distally in each cell (purple bar) and Ft (blue bar) is proximal. In prepupal and pupal wings, Fj expression is maintained in a radial gradient from the wing margin (yellow), and Ds is expressed in the centre of the wing (purple). Ft and Ds maintain a radially polarized pattern of subcellular localization in prepupal and 28 h APF pupal wings. (*c*) Clones of cells lacking Fz activity have non-cell-autonomous effects on neighbouring wild-type tissue. Trichomes normally point distally (black arrows). In *fz* mutant cells (grey), the Stbm (orange) in cells at the clone edge localizes to the clone boundary, where it can form asymmetric complexes with Fz (green) in wild-type cells. This causes alterations in trichome polarity (red arrows) in wild-type tissue next to lateral and distal clone edges. Self-organizing feedback interactions lead to propagation of this aberrant polarity across several cells. Proximal is left and distal is right. (*d*) Schematics showing the direction of non-autonomy next to core pathway clones in the wing. Clones are depicted as loss of green or orange colour, and trichomes normally point distally (black arrows). Clones of cells lacking Fz activity (left) have non-autonomous effects on trichome orientation on the distal side of the clone (red arrows), while clones lacking Stbm activity (right) affect trichomes on the proximal side of the clone (red arrows). Trichomes always point away from cells with higher Fz activity, and the direction of non-autonomy is, therefore, a read out of Fz localization. Overexpression clones have opposite effects to loss-of-function clones (not shown). (*e*) Schematics showing non-autonomy (red arrows) proximal to clones of cells lacking Ft activity (loss of blue colour) and distal to clones of cells lacking Ds activity (loss of purple colour) in the wing. The extent of non-autonomy is weaker and more variable for Ft–Ds pathway clones than for core pathway clones.

The pupal wing derives from the larval wing imaginal disc, which during pupal stages everts and folds to form a double-layered epithelium with the two basal surfaces apposed (figure 2*a*). It consists of thousands of cells, each of which forms a single distally pointing trichome (figure 1*a*). The seven-pass transmembrane protein Frizzled (Fz) localizes to distal cell ends, together with the cytoplasmic proteins Dishevelled (Dsh) and Diego (Dgo) [9–13]. The four-pass transmembrane protein Strabismus (Stbm, also known as Van Gogh [Vang]) and the cytoplasmic protein Prickle (Pk) localize to proximal cell ends [14,15]. Finally, the atypical cadherin Flamingo (Fmi, also known as Starry Night [Stan]) localizes to both proximal and distal cell ends, and mediates homophilic intercellular interactions (figures 1*d*,*f* and 2*a*) [16]. Loss of any of the core complex components results in a loss of planar polarity, with trichomes initiating from the centre of the cell and forming a characteristic swirling pattern across the surface of the wing epithelium [10,16–20].

The core proteins are known to regulate trichome positioning in the wing via a group of ‘effector’ proteins: Inturned (In), Fuzzy (Fy), Fritz (Frtz), Rab23 and Multiple Wing Hairs (Mwh) [17,21–24]. While this process is not fully understood, asymmetric localization of the core proteins leads to proximal localization of In, Fy and Frtz and In-Fy have recently been found act as a GDP-GTP exchange factor (GEF) complex for Rab23. This leads, by an unknown mechanism, to a proximal-to-distal gradient of Mwh localization [24–27]. Mwh encodes a formin-homology 3 (FH3) domain protein, that inhibits actin polymerization, and restricts formation of the actin-rich trichome to the distal cell edge [26–28].

### 2.2. The core pathway in the *Drosophila* eye

**Figure 3.**
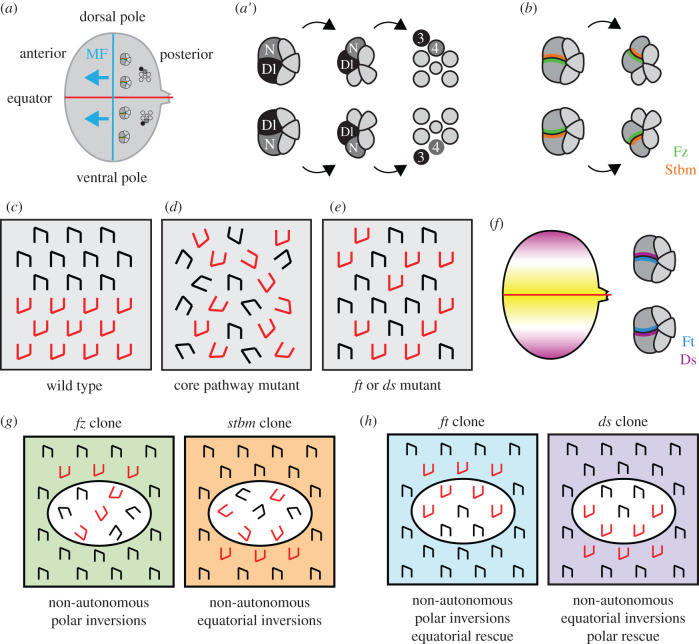
Planar polarity in the *Drosophila* eye. (*a*) Ommatidial orientation in the eye imaginal disc, where (*a*′) is zoomed in. (*a*) The dorsal pole of the eye is up and the ventral pole is down, and the red line shows the dorsoventral midline (equator). The blue line shows the morphogenetic furrow, a wave of differentiation that passes from the posterior to the anterior (blue arrows), with photoreceptor cells being progressively added to the ommatidial clusters. (*a*′) As photoreceptor differentiation proceeds clusters in the dorsal half of the eye rotate clockwise, and those in the ventral half of the eye rotate anti-clockwise, and the clusters acquire opposite chiralities. Notch (N) activity is high in the R4 photoreceptor cell and its ligand Delta (Dl) is high in R3. (*b*) Core protein localization in the eye imaginal disc. At the five-cell stage, Fz (green) localizes to the polar boundary of the equatorial cell, that will become R3. Stbm (orange) localizes to the equatorial boundary of the polar cell, that will become R4. (*c*) Schematic showing ommatidia on either side of the equator with mirror-image symmetry. Ommatidia with opposite chiralities are shown in black or red. (*d*,*e*) Schematics showing ommatidial orientation in eyes lacking core pathway (*d*) or Ft–Ds pathway (*e*) activity. (*d*) In core pathway mutants, ommatidia have randomized chirality and rotation. (*e*) In Ft–Ds pathway mutants, ommatidia rotate the correct amount, but have randomized chirality. This is indicative of an incorrect R3/R4 fate decision. (*f*) Ft–Ds pathway expression and localization in the eye imaginal disc. Ds (purple) is expressed in a gradient that is high at the poles, while Fj expression (yellow) is highest at the equator. Ds localizes to equatorial cell boundaries (purple), while Ft is inferred to localize to polar cell boundaries (blue). (*g*) Schematics showing the direction of non-autonomy next to core pathway clones in the eye. Clones are shown in the dorsal half of the eye, where all ommatidia should have dorsal chirality (black). Clones of cells lacking core pathway activity have randomized chirality and rotation inside the clone. Clones lacking Fz activity cause inversions of wild-type ommatidia on the polar sides of clones (red ommatidia outside the clone), while ommatidia on the equatorial side of the clone are inverted outside clones lacking Stbm activity. (*h*) Schematics showing the direction of non-autonomy next to Ft–Ds pathway clones in the eye. Wild-type ommatidia on the polar side of *ft* clones are inverted, and there is rescue of mutant ommatidia on the equatorial clone boundary. *ds* clones cause inversions of wild-type ommatidia on the equatorial side of clones, and there is polar rescue of mutant ommatidia.

The *Drosophila* eye consists of approximately 800 facets or ommatidia, each of which contains a cluster of around 20 photoreceptors and support cells (figure 1*b*). Photoreceptors are specified in the epithelium of the larval eye imaginal disc, where a wave of differentiation (the morphogenetic furrow) passes from posterior to anterior. As the photoreceptors differentiate the clusters are initially symmetric, but the clusters gradually rotate 90° and become asymmetric. Ommatidial clusters on either side of the dorsoventral midline (equator) rotate in opposite directions and thus acquire opposite orientation (figure 3*a*,*c*) [29]. Ommatidial orientation and direction of rotation are regulated by Notch signalling between the R3 and R4 photoreceptor cells, whereby the cell with higher Notch activity takes on the R4 cell fate [30–32].

Notch signalling is biased by the asymmetric localization of the core proteins. Fz and Dsh localize to the polar edge of the R3 cell and promote R3 cell fate, while Stbm localizes to the equatorial edge of the R4 cell and promotes R4 cell fate (figure 3*b*) [30–36]. R3 cell fate specification has been suggested to be a result of a direct interaction between Dsh and the intracellular domain of Notch, that inhibits Notch activity [36]. Alternatively, Fz has been proposed to upregulate expression of the Notch ligand Delta via the Jun transcription factor [37–39] and to increase Delta activity via upregulation of the E3 ubiquitin ligase Neutralized [40]. Fz also upregulates transcription of the small GTPase Ral in R3, where Ral inhibits Notch activity [41].

In addition to controlling ommatidial orientation via the R3/R4 fate decision, the core pathway regulates the degree of rotation (figure 3*d*) [10,33–35,42]. This is thought to occur via cytoskeletal regulators. These include signalling by the small GTPases RhoA and Rac, upstream of Rho kinase [36,43–45] and modulation of E-cadherin and N-cadherin expression via the kinase Nemo [46–49]. Notch activity in the R4 cell also contributes to rotation by regulating EGF signalling [46,50–53].

### 2.3. The core pathway in the *Drosophila* abdomen

**Figure 4.**
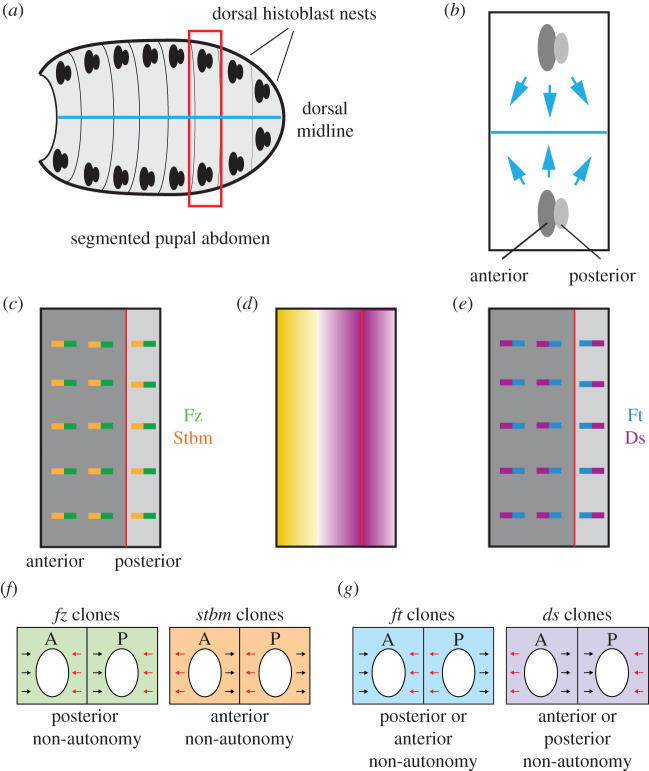
Planar polarity in the *Drosophila* abdomen. (*a*,*b*) Schematics of abdomen development. (*a*) The adult abdomen derives from histoblast nests that are specified in the embryo. Each segment of the pupa contains 2 pairs of dorsal histoblast nests (anterior and posterior, black ovals), one pair on each side of the dorsal midline (blue line), as well as pairs of ventral nests and spiracular nests (not shown). (*b*) Close-up of the region outlined in red in (*a*). During pupal development, the histoblast nests proliferate and migrate dorsally to cover the whole dorsal surface of the abdomen, replacing the larval epithelial cells that undergo apoptosis. (*c*) Core protein localization in the abdomen. Stbm (orange) localizes to anterior cell edges in both anterior and posterior compartments, while Fz (green) is inferred to localize to posterior cell edges. (*d*) Ft–Ds pathway expression in the abdomen. Ds (purple) expression is high at the boundary between the anterior and posterior compartments, while Fj (yellow) has highest expression at the anterior of the anterior compartment. (*e*) Ft–Ds localization in the abdomen. Ds (purple) and Dachs (not shown) are localized to the anterior cell edges in the anterior compartment and posterior cell edges in the posterior compartment. Ft (blue) is thought to be localized to opposite cell edges. (*f*) Schematics showing the direction of non-autonomy next to core pathway clones in the abdomen. Clones are depicted as loss of green or orange colour, and hairs normally point posteriorly (black arrows). Clones lacking Fz activity cause wild-type hairs posterior to the clone to point towards the clone (red arrows), while clones lacking Stbm activity cause wild-type hairs anterior to the clone to point away from the clone (red arrows). Clones behave the same way in both compartments. (*g*) Schematics showing non-autonomy (red arrows) next to clones lacking Ft or Ds activity (loss of blue or purple colour) in the abdomen. Clones of cells lacking Ft or Ds have opposite effects, and clones also have opposite effects in anterior and posterior compartments.

The adult abdomen is a segmented epithelial structure where the cells form hairs and bristles that point posteriorly (figure 1*c*). The dorsal abdominal epithelium develops from two anterior and posterior histoblast nest pairs per segment, located on either side of the dorsoventral midline (figure 4*a*,*b*). During pupal development these histoblast nests divide and migrate before fusing together at the dorsoventral midline and anteroposterior segment boundaries, to form a continuous epithelium that displaces the larval epithelium (figure 4*b*,*c*). Molecular aspects of core protein localization and activity are less well examined than in other tissues, but Stbm is known to localize to anterior cell edges (figure 4*c*) [54], and loss of core protein activity disrupts hair and bristle polarity [17,55]. The same effector proteins that control trichome orientation in the wing (In, Fy, Frtz and Mwh) also appear to regulate hair polarity in the abdomen [17,22,56].

### 2.4. Self-organization of the core proteins

The asymmetric cellular localization of the core proteins is thought to be driven by a global cue that provides a small bias in asymmetry across the tissue axis. This initial bias is then amplified by self-organizing feedback interactions between the core proteins themselves, whereby intercellular complexes of the same orientation are locally stabilized and those of opposite orientation are destabilized (figure 1*h*). Mathematical modelling has confirmed that feedback interactions are a plausible mechanism for sorting complexes into a uniform orientation, and can thus amplify an initial bias in asymmetry (e.g. [57–60]). The existence of feedback interactions has also been supported by recent experimental evidence (e.g. [61,62]). However, how the initial bias is achieved, such that complexes align with respect to the tissue axis, is less well understood, and will be discussed later.

### 2.5. Non-cell-autonomous effects of the core pathway

A key feature of core pathway function in all these tissues is that groups of cells (clones) lacking Fz or Stbm activity cause non-cell-autonomous effects, such that the polarity of wild-type tissue adjacent to the clone is perturbed. In the wing, trichomes distal to *fz* clones point back towards the clone [63]; while trichomes proximal to *stbm* clones point away from the clone (figure 2*d*) [19]. The opposite effect is seen for clones overexpressing Fz or Stbm [12,57,64]. This behaviour is thought to be due to the self-organizing feedback interactions between the core proteins on the clone boundary [57–60,65]. Mutant cells on the boundary of *fz* clones accumulate Stbm on the clone boundary, as this is the only site of interaction with Fz in neighbouring cells. This inverts polarity on the distal side of the clone, and the effect is propagated over several cells (figure 2*c*). This demonstrates that Fz and Stbm are required not only to polarize individual cells (intracellular signalling), but also to communicate polarity information between neighbouring cells (intercellular signalling). Within the clone, the trichome emerges from the centre of the cell due to a failure in intracellular feedback and loss of asymmetric protein localization [18]. Outside the clone, in contrast, the trichome emerges from an aberrant cell edge, as intracellular feedback is operational but intercellular signalling is disrupted [66–69].

Similar reciprocal relationships for loss- or gain-of-function clones of *fz* and *stbm* are seen in the eye and abdomen (figures 3*g* and 4*f*) [33,55,67]. In particular, wild-type ommatidia on the polar side of *fz* clones are rotated the correct amount, but have an inverted orientation, while ommatidia on the equatorial side of *stbm* clones are inverted (figure 3*g*) [33,67]. Thus, ommatidial inversions in the eye are a hallmark of a disruption in intercellular communication of polarity information.

## 3. The Ft–Ds pathway in *Drosophila*

Ft and Ds are atypical cadherins with 34 and 27 cadherin repeats, respectively [70,71]. Like the core proteins, they form heterophilic intercellular complexes between adjacent cells in fly epithelial tissues [72,73] and localize asymmetrically (figure 1*e*,*g*) [74–77]. The atypical myosin Dachs colocalizes with Ds at cell junctions [74–76,78]. Heterophilic binding between Ds and Ft is regulated by the Golgi-localized kinase Four-jointed (Fj): Fj phosphorylates the extracellular cadherin repeats of Ft and Ds. This enhances the ability of Ft to bind to Ds, but reduces the ability of Ds to bind to Ft [79–83]. Similarly to the core proteins, Ft and Ds are recruited to the boundaries of clones mutant for each other, and to boundaries of clones overexpressing Ds or Fj. This can lead to propagation of aberrantly oriented complexes for several cell diameters [66,72,74,76].

Asymmetric localization of Ft and Ds is driven by opposite expression gradients of Ds and Fj (figure 2*b*). In the wing disc, Fj is expressed in a gradient, high distally and low proximally [79,84,85], while Ds is highly expressed in the proximal hinge region [71–73,85]. In the pupal wing, Fj expression is higher at the wing margin [72,73,79,86], while Ds expression extends in a finger from the hinge, along the centre of the wing [73,87,88]. Ft appears to be uniformly expressed [66,70,89]. These expression patterns lead to opposing gradients of Ft and Ds binding affinities, and modelling predicts that these opposing gradients are sufficient to generate asymmetry such that Ds localizes to distal cell edges and Ft localizes to proximal cell edges [74,76,82,83].

Similar complementary expression patterns of Fj and Ds are seen in the eye and abdomen, and pathway components are again asymmetrically localized (figures 3*f* and 4*d*,*e*) [74,76,77,79,84,86,90–92]. Like the core pathway, loss-of-function mutations in *ft*, *ds* and *fj* disrupt hair polarity in the wing and abdomen [66,72,86,91,93], while mutant ommatidia have polarity inversions but are rotated the correct degree (figure 3*e*) [90,92,94,95]. However, loss of Dachs activity has only mild effects on ommatidial orientation and hair polarity [78,96], despite the fact that it is asymmetrically localized with Ds.

In addition to affecting the planar polarity of cuticular structures, Ft and Ds also regulate growth via the Hippo signalling pathway, as well as tissue shape. Wings with reduced Ft or Ds activity are shorter and rounder than normal, and effects on both growth and tissue shape are mediated by Dachs. In growth regulation, Ft inhibits Dachs activity, which in turn inhibits the activity of the kinase Warts (Wts). Wts phosphorylates and negatively regulates the transcription factor Yorkie (Yki). In the absence of Ft activity, Yki activates target genes, leading to tissue overgrowth (for more details, see [97–99]). Consistent with Dachs and Wts being key mediators of the growth control function of Ft, loss of Dachs or overexpression of Wts can rescue the overgrowth seen in *ft* mutants [78,85,100,101].

Acting downstream of Ft and Ds, Dachs asymmetric localization at cell junctions also controls polarized tension and cell shape [75,102,103], and loss of Dachs results in shorter, narrower wings. One result of Dachs asymmetry is oriented cell divisions [102,104,105]. However, surprisingly, wing elongation is normal in the absence of oriented cell division [106], suggesting that the ability of Dachs to regulate junctional tension has additional roles in regulating wing shape.

## 4. Interactions between Ft–Ds and the core proteins

The data above demonstrate that both Ft–Ds and the core proteins act in multiple tissues on the same axes to polarize various cellular structures. This suggests two models. First, they could be acting independently, and both pathways are needed for the final polarity decision. Alternatively, they could be acting sequentially to specify the final polarity.

### 4.1. Evidence for sequential action of Ft–Ds and the core proteins

Experiments in the eye suggest a sequential mode of action, in which Ft–Ds gradient cues provide dorsoventral polarity information upstream of core pathway activity. *ft*, *ds* and *fj* clones cause non-autonomous inversions of ommatidial polarity on clone boundaries in the eye, similar to *fz* and *stbm* clones (figure 3*g*,*h*) [90,92,94–96]. Non-autonomous inversions of polarity propagate from the polar edge of *ft* clones, and there is a corresponding rescue of polarity of mutant ommatidia on the opposite side of the clone (figure 3*h*) [92,94–96]. This rescue of mutant tissue by neighbouring wild-type tissue argues against propagation of Ft–Ds complexes directly regulating ommatidial orientation. However, the core proteins remain asymmetrically localized in *ft* or *ds* mutant tissue, but core protein asymmetry is randomized [92]. This leads to a simple model whereby dorsoventral gradients of Ds and Fj in the eye result in asymmetric localization of Ft–Ds, and this then directs the orientation of core protein asymmetric localization. The core proteins then interact with Notch to bias the R3/R4 fate decision and with downstream effectors to regulate the degree of rotation.

Early experiments in the wing were largely consistent with such a model of sequential action. In the absence of *ft* or *ds* the core proteins localize asymmetrically, but in the incorrect orientation, and this leads to corresponding defects in trichome polarity [66,72,73]. *ft* and *fj* clones show variable proximal non-autonomy in some regions of the wing, and *ds* clones show weak distal non-autonomy (figure 2*e*) [66,72,86,93]. Moreover, non-autonomy around *fz* clones extends further in a *ds* or *ft* mutant background, consistent with loss of an upstream cue that would normally antagonize aberrant propagation of core protein complex asymmetry [72,93,107]. Thus, it was suggested that graded expression of Ds and Fj in the wing gives directional information to the core proteins, and local cell–cell communication via the core proteins then allows robust cell-to-cell propagation of polarization [72].

### 4.2. Evidence against a sequential action model

**Figure 5.**
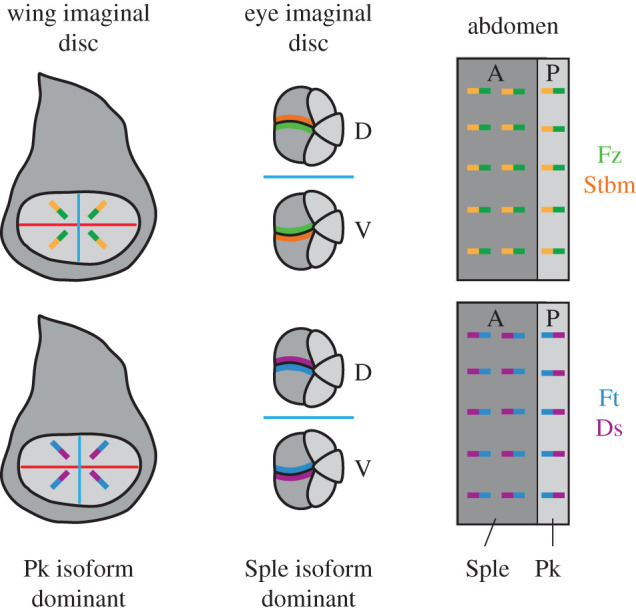
Asymmetric localization of polarity proteins and Pk isoform expression in the wing, eye and abdomen. In the wing imaginal disc and posterior compartment of the abdomen, Stbm (orange) and Ds (purple) are localized to opposite cell edges, and the Pk^Pk^ isoform is dominant. In the eye imaginal disc and anterior compartment of the abdomen, Stbm and Ds localize to the same cell edges, and the Pk^Sple^ isoform is dominant.

Despite the findings described above, a number of observations in the wing and abdomen have challenged the idea that the Ft–Ds and the core proteins act sequentially in all tissues and at all times:

(1) In the eye and anterior compartment of the abdomen, Fz and Ds are localized on opposite cell edges; while in the wing and posterior compartment of the abdomen they are on the same cell edges (figure 5). If Ft–Ds directly regulate core protein localization, the relative orientation might be expected to be constant. This will be further discussed in §5.
(2) Ft and Ds gradients do not seem to be important for orienting trichomes in most of the wing. Importantly, uniform overexpression of Ds, or Ds and Fj together, can almost completely rescue the mutant trichome orientation defect [73,108,109]. Likewise, *ft* mutant phenotypes can be rescued by overexpression of the intracellular domain of Ft, which neither binds to Ds nor localizes asymmetrically [109–111]. Finally, blocking overgrowth in *ft* mutants, for example by overexpression of Wts or loss of Dachs, also rescues trichome orientation [76,101,112]. In all these cases any remaining trichome orientation defects are restricted to the proximal wing. This suggests that Ft and Ds activity does not have to be localized subcellularly in order for trichomes to polarize correctly over most of the wing (see §6.1).
(3) The localization patterns of the core proteins and Ft–Ds in the developing wing diverge over time. Asymmetric localization of the core proteins has first been reported in the third instar larval imaginal disc [76,113,114]. As the wing disc everts and undergoes morphogenetic movements, core protein asymmetry is at least partially preserved. In early pupal wings, asymmetry is initially directed towards the dorsoventral boundary, but is then rearranged to proximodistal as the wing extends along this axis (figure 2*a*) [113,115]. This rearrangement is thought to be a result of tissue flows—distinct patterns of oriented cell division, cell elongation and cell rearrangements—caused by hinge contraction [115]. Ft and Ds localize similarly to the core proteins in the wing disc and early pupal wing (figure 2*b*) [74,76,88,116]. However, at the time that the core proteins realign on the proximodistal wing axis, Ds remains localized towards the wing margin [88]. In particular, Ft–Ds continue to align to the Ds and Fj gradients, where Fj is around the wing margin and Ds extends in a finger along the centre of the wing (figure 2*b*) [72,73,79,86–88]. Thus at the time of trichome emergence, Ft–Ds are orthogonal to the core over much of the wing blade (figure 2*a*,*b*), and this indicates that Ft–Ds polarity cannot be directly coupled to the core proteins at this stage of wing development (see §6.2).
(4) Ft and Ds can independently control polarity in the abdomen. Clones of cells overexpressing Ft can reverse the polarity of hairs in adjacent wild-type tissue, even in the absence of Fmi or Fz activity [107,117]. If Ft and Ds were strictly upstream of the core proteins, then Ft–Ds would not be able to repolarize hairs in their absence (see §7).

## 5. Coupling of Ft and Ds to the core proteins via Pk^Sple^

The story so far suggests that Ft–Ds and the core proteins may be coupled at some times and places, but not at others. What might be the basis for this selective behaviour? One answer to this has come from studies of the cytoplasmic core protein Pk.

### 5.1. Pk isoform expression correlates with the relative orientation of Ft–Ds and core protein complexes

As well as being a component of the core pathway that is required for intracellular amplification of asymmetry, Pk also has additional roles in coupling the core pathway to upstream cues, specifically Ft–Ds. Pk has two functional splice forms: a short version which is known as the Pk^Pk^ isoform and a version with a longer N-terminus known as Pk^Spiny-legs^ (Pk^Sple^) [42]. The unique N-terminus of Pk^Sple^ can physically interact with Ds [118,119]. Pk^Sple^ also interacts with Dachs [118,119], although this interaction is likely to be less important, as loss of Dachs results in negligible planar polarity defects [78,96]. This suggests that Ft–Ds and core protein asymmetry could be directly coupled within cells in the presence of Pk^Sple^.

The Pk^Pk^ and Pk^Sple^ isoforms appear to be differentially active in different tissues, with mutation of the *pk^sple^* isoform giving a polarity phenotype in the eye, leg and anterior abdomen; and mutation of the *pk^pk^* isoform giving a polarity phenotype in the wing and posterior abdomen [17,42,88,118–121]. Thus, in the eye the Pk^Sple^ isoform localizes with Stbm to the equatorial edge of the R4 cell and in the anterior compartment of the abdomen Pk^Sple^ localizes with Stbm to anterior cell edges (figure 5) [119]. Intriguingly, Ft–Ds and the core proteins share the same relative orientation in tissues in which Pk^Sple^ is the predominant isoform (the eye and anterior compartment of the abdomen), while they have the opposite relative orientation in tissues in which the Pk^Pk^ isoform is predominant (the wing and posterior compartment of the abdomen) (figure 5). This led to the suggestion that Pk activity can ‘rectify’ or reverse the direction of coupling of polarity between Ft–Ds and the core proteins [55].

### 5.2. Gradients of Ft and Ds activity regulate the core proteins via Pk^Sple^

In the eye, the evidence supports a model in which Ft–Ds act strictly upstream of the core proteins, and the two pathways are directly coupled via Pk^Sple^. In the absence of Pk^Sple^, ommatidia are rotated 90° but have randomized dorsoventral polarity [120]—the same phenotype as loss of Ft and Ds [92,94,95]. Dachs, Stbm and Pk^Sple^ all localize to the equatorial edge of the R4 cell (figure 5) [36,76,119], and the direction of non-autonomy of mutant clones supports coupling between Ds-Dachs and Pk^Sple^-Stbm (figure 3*g*,*h*) [33,67,90,92,94,96]. Interestingly, while the Ds and Fj expression gradients are not essential for trichome orientation in the wing [73,108,109], uniform expression of Ds and Fj causes a complete randomization of ommatidial chirality [108], and reversing the gradient of Fj activity reverses ommatidial polarity [90]. Thus a model emerges whereby gradients of Ds and Fj lead to asymmetric localization of Ft–Ds–D, which couples to the core proteins via Pk^Sple^ to direct the orientation of asymmetric localization of the core proteins and ommatidial rotation.

Likewise, planar polarity in the anterior compartment of the abdomen is dependent on the Pk^Sple^ isoform [17,119]. Stbm, Pk^Sple^ and Dachs all localize to anterior cell ends (figure 5) [54,119] and clones of cells lacking Stbm or Ds exhibit non-autonomy in the same direction (figure 4*f*,*g*) [55,91], suggestive of direct coupling of the core proteins to Ft and Ds via Pk^Sple^. Consistent with Pk^Sple^ being a key mediator between Ft–Ds and the core proteins in the anterior compartment of the abdomen, *fz* non-autonomy extends further in the absence of Pk isoforms in this compartment [55], as it does in the absence of Ds [107].

Pk^Sple^ activity is not required in the posterior compartment of the abdomen, where Pk^Pk^ is dominant, and Pk^Pk^ and Dachs localize to opposite cell edges (figure 5) [119]. Loss of Pk^Pk^ causes hair reversals, but this is not seen if both isoforms are absent [55,119]. Importantly, this result shows that the reversals of polarity in the posterior compartment in the absence of Pk^Pk^ are dependent on the weakly expressed Pk^Sple^ isoform. Indeed, overexpressing Pk^Sple^ in the posterior abdomen also reverses hair polarity [54,55], and this overexpressed Pk^Sple^ localizes to posterior cell edges—the same as Dachs [119]. Therefore, Pk^Sple^ is able to ectopically couple the core proteins to Ft–Ds when expressed in the posterior compartment.

Like the posterior abdomen, the wing does not normally require Pk^Sple^ activity for correct planar polarity to be established. However, loss of Pk^Pk^ in the wing produces a swirling pattern of trichomes that is distinct from the characteristic ‘core phenotype’, such that trichomes point towards the centre of the wing [17,42]. Overexpression of Pk^Sple^ causes a similar trichome swirling phenotype [42,122–124], suggesting that, as in the posterior abdomen, the phenotype of *pk^pk^* mutants is dependent on Pk^Sple^ activity.

When Pk^Sple^ is overexpressed, it localizes distally or anteroposteriorly rather than proximally in each cell over much of the wing, to cell edges opposite to the site of mispolarized trichome formation [54,88,118,119,125]. Thus, the core protein complex is still asymmetrically localized but it no longer orients along the proximodistal cell axis. In fact, patterns of core protein asymmetric localization in *pk^pk^* pupal wings closely correlate with patterns of Ds localization [88]. Crucially, the trichome phenotype and mislocalization of the core proteins seen in *pk^pk^* mutants or when Pk^Sple^ is overexpressed appears to be dependent on Ft and Ds. If Ft–Ds activity is reduced, trichomes no longer point towards the centre of the wing, and misexpressed Pk^Sple^ is unpolarized or proximally localized [87,88,118,119]. This fits a model whereby Pk^Sple^ ectopically couples to Ft–Ds when it is overexpressed or in the absence of Pk^Pk^, and this reorients core protein localization to align with Ft–Ds polarity.

**Figure 6.**
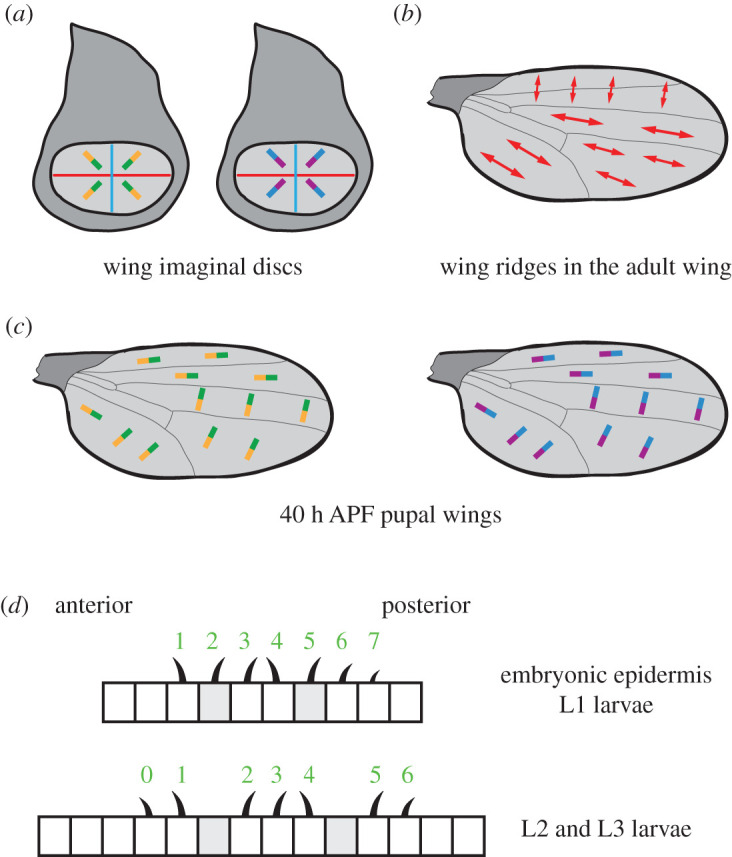
Regulation of wing ridge polarity and denticle polarity. (*a*) Third instar wing imaginal discs. Stbm (orange) and Ds (purple) are aligned in opposite orientations. (*b*) Orientation of wing ridges in adult wings. Adult wings consist of a transparent cuticle that is secreted by wing epithelial cells during pupal development. In addition to each cell producing a distally pointing trichome, the surface of the wing cuticle is also ridged. Wing ridges (red arrows) run proximodistal in the posterior of the wing, and anteroposterior in the anterior wing. (*c*) 40 h APF pupal wings. Stbm (orange) and Ds (purple) are aligned in the same orientation. (*d*) Denticle polarity in embryos and first instar larvae (L1, top) and in second and third instar larvae (L2 and L3, bottom). A subset of cells in each segment secrete denticles, most of which point posteriorly. Rows 1 and 4 in the embryo and rows 0, 1 and 4 in L2 and L3 point anteriorly. Tendon cells are shaded in grey.

Studies on adult wing ridges also support Pk^Sple^-mediated coupling between Ft–Ds and the core proteins (figure 6*a*–*c*). The adult wing is a ridged transparent cuticle, where ridges run proximodistal in the posterior wing and anteroposterior in the anterior wing (figure 6*b*) [123]. Ridge orientation is dependent both on core protein activity and Ft–Ds activity [87,88,123]. *pk^pk^* mutants largely affect anterior ridge orientation, while *pk^sple^*, *ft* or *ds* mutants largely affect posterior ridge orientation. Specification of wing ridges coincides with an increase in Pk^Sple^ expression at 40 h APF [88,121], and a further rearrangement of both core protein and Ft–Ds localization (figure 6*c*). Molecular and genetic evidence are consistent with Ft–Ds regulating the core proteins via Pk^Sple^ in the posterior wing [88].

In summary, the evidence suggests that Ft–Ds direct the asymmetric localization of the core proteins via Pk^Sple^ in the eye, anterior compartment of the abdomen and during posterior wing ridge development. In the posterior compartment of the abdomen and during trichome specification in the wing, there is so far no evidence for a direct link between Ft–Ds and the core proteins, but Pk^Sple^ can ectopically couple the two pathways if it is overexpressed or if Pk^Pk^ is absent.

This does not, however, imply that that Ft–Ds can only couple to the core pathway through Pk^Sple^. Remaining questions include the following. Can Ft–Ds influence the core via other mechanisms? Can Ft–Ds act independently of the core? What other cues input to the core pathway when Ft–Ds are not active? We will elaborate on these themes in the next sections by discussing some tissue-specific instances.

## 6. Indirect effects of Ft and Ds on core protein localization

As discussed above, Pk^Pk^ is the dominant isoform in the wing at the time of trichome formation, suggesting that the core proteins must become oriented in response to cues independently of Pk^Sple^ coupling to Ft–Ds. Nevertheless, loss of Ft and Ds does affect trichome orientation [66,72,91,93]. How can this be explained? In this and the following section we discuss two classes of mechanism that appear to act in different regions of the wing.

### 6.1. Disruption of trichome orientation downstream of Ft activity

Notably, trichome polarity defects in *ft* and *ds* mutants are largely abolished if signalling pathways downstream of Ft are mutated (as described in §4.2) [76,101,109–112]. This suggests that Ft and Ds indirectly affect trichome orientation via Ft signalling. Loss of Ft or Ds causes overgrowth via Hippo signalling [97–99], and so one possibility is that this overgrowth disrupts the response of Fz and Stbm to non-Ft–Ds-dependent upstream cues. Alternatively, loss of Ft causes mislocalization of Dachs [78], and this could disrupt Pk^Sple^ localization [119]. In addition loss of Ft and Ds disrupts cell packing and this has been proposed to result in propagation of core protein polarity in aberrant directions [126]. Another mechanism could be disruption of tissue reorganization by Ft and Ds. As discussed earlier, core protein asymmetry is directed towards the dorsoventral boundary in the early pupal wing, but is then rearranged to proximodistal as the wing extends along this axis during hinge contraction (figure 2*a*) [113,115]. The tissue flows that are believed to cause the rearrangement of core protein localization are disrupted in the absence of Ft or Ds [115], and this could impact on core protein localization. To conclude, loss of Ft–Ds could disrupt trichome orientation indirectly via alterations in cell division and cell behaviours, and disruption of tissue architecture, any of which could disrupt the ability to respond to upstream polarity cues or create mismatches in polarity between neighbouring cells.

### 6.2. Regulation of microtubule orientation by Ft and Ds

When Ft–Ds activity is lost but overgrowth is suppressed, trichome polarity defects are still present in the proximal wing [76,101,109–112]. In keeping with this, asymmetric localization of the core proteins in the proximal wing disc is disturbed in *ft* or *ds* mutants [76,114]. Hence, Ft–Ds do seem to provide some directional input in this region of the wing. Both Ft–Ds and core protein complexes form discrete puncta in the cell junctions of wing disc cells, but these puncta do not extensively co-localize [88], arguing against the presence of protein–protein interactions between components of the two pathways. Moreover, the core proteins are not recruited to *ft* or *ds* clone boundaries in the wing [66], which would be expected if Ft–Ds and the core proteins show strong direct interactions. Hence a more indirect mechanism might be in play.

**Figure 7.**
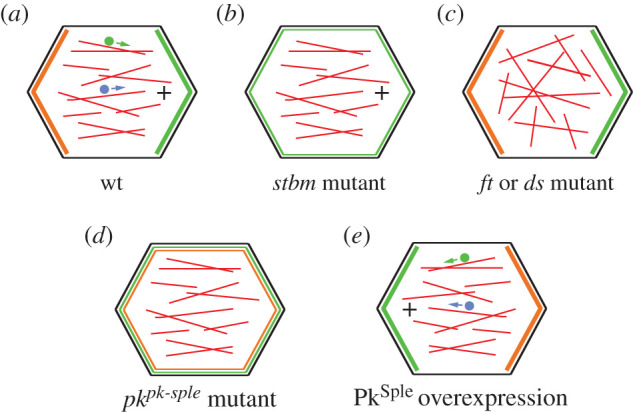
Regulation of microtubule alignment and polarity. (*a*) In wild-type wings at 24 h APF, microtubules are aligned along the proximodistal axis, and in the proximal region of the wing the plus ends are subtly biased towards the distal cell end. Fz and Dsh particles (green and blue dots, respectively) are associated with microtubules and their movement is biased towards microtubule plus ends. (*b*) Loss of Fz or Stbm does not affect microtubule alignment or plus-end bias. (*c*) Loss of Ft or Ds disrupts microtubule alignment along the proximodistal axis. (*d*) Loss of both isoforms of Pk does not affect microtubule alignment, but the plus-end bias in the proximal wing is lost. (*e*) Overexpression of Pk^Sple^ reverses the bias in microtubule plus ends in the proximal region of the wing, and there is a reversal in the movement of Dsh particles.

One mechanism by which Ft and Ds have been suggested to influence core protein localization in the proximal wing is via polarized transport of the core proteins on microtubules. Microtubules are aligned along the proximodistal axis of the wing from third instar stages, and in the proximal region of the wing there is a subtle bias of microtubule plus ends towards the distal end of the cell (figure 7*a*) [116,127,128]. Fz-containing vesicles have been observed on microtubules, and both Fz and Dsh move along the proximodistal axis with a slight distal bias [54,116,127]. This polarized transport has been proposed to promote Fz and Dsh localization to distal cell ends.

Microtubule alignment is decreased in *ft* and *ds* mutants, the distal plus-end microtubule bias in the proximal wing is lost and Dsh particles move without directional bias and with less processivity (figure 7*c*) [54,116,128]. This leads to a model whereby Ft–Ds influence microtubule alignment in the proximal wing and this biases Fz localization to distal cell ends. This initial bias could then be amplified by local feedback interactions (figure 1*h*).

Ft–Ds may also regulate microtubule orientation and core protein polarity in the anterior compartment of the abdomen. Here, microtubule plus-end growth is weakly biased towards the posterior ends of cells [54], although predominantly growth is along the mediolateral axis of the abdomen, orthogonal to the axis of asymmetry [129]. Consistent with this slight plus-end bias, Dsh particles show a posterior bias in their transport, as expected from the axis of the polarity of the core proteins [54]. Plus-end bias is lost in *ft* mutants where overgrowth is suppressed [130]. However, microtubule plus ends are oppositely oriented in the posterior compartment and fewer Dsh particles are seen [130], which suggests that microtubules are not important for transport of Dsh in this compartment.

The mechanism by which Ft–Ds could bias microtubule alignment is not clear, but has been suggested to involve the serine-threonine kinase PAR-1 [128]. Another possibility is suggested by the observation that microtubules align on the longest axis of the cell in the pupal wing epithelium, the embryonic epidermis and the follicular epithelium [131]. Ft–Ds can affect cell shape via Dachs in the wing disc or the pupal notum [75,102], and therefore the effect of Ft–Ds on microtubule alignment could be an indirect effect of modulating cell shape. Other work has suggested microtubule stability is independent of cell shape: Ft and Ds were proposed to stabilize microtubules at proximodistal adherens junctions [116,132], and microtubules that interact with the proximodistal cell junctions have a longer lifetime than those that interact with the anteroposterior cell junctions [132]. Further detailed analysis of cell shape and microtubule orientation will be needed to examine these hypotheses.

Interestingly, Ft and Ds regulate microtubule alignment in third instar imaginal discs [116], and core protein localization is also perturbed in proximal regions of *ft* and *ds* mutant wings in third instar imaginal discs [76,114]. This suggests that the microtubule-based transport of Fz and Dsh is an early event in the development of wing cell polarity. Such an early role for Ft–Ds in regulating the core proteins could explain the later divergence in localization patterns (see §4.2). Thus Ft–Ds may be required to regulate core protein polarity via microtubule-based mechanisms in the early proximal wing region, but core protein polarity is subsequently rearranged from radial to proximodistal by tissue flows independently of this mechanism.

### 6.3. Pk isoforms regulate microtubule plus-end bias downstream of Ft and Ds

Intriguingly, Pk isoforms also regulate the direction of the microtubule plus-end bias in the proximal wing. Loss of both isoforms of Pk abolishes plus-end bias (figure 7*d*), but re-expression of Pk^Pk^ is sufficient to restore plus-end bias [54]. Moreover, overexpression of Pk^Sple^ in the wing reverses the microtubule plus-end bias and Dsh vesicle movement (figure 7*e*) [54]. However, the effects on microtubule orientation and core protein localization appear to be independent. Pk^Sple^ overexpression reverses microtubule polarity only in the proximal wing [54,130], while trichome polarity is reversed over the entire wing [42,122–124]. Furthermore, loss of Fz or Stbm does not affect plus-end bias (figure 7*b*) [54,128], and microtubule plus-end bias can be reversed by overexpression of Pk^Sple^ in a *stbm* mutant [54]. Interestingly, in fly axons, Pk^Pk^ promotes microtubule minus-end bias toward the cell body, while Pk^Sple^ promotes bias towards the synapse [133]. This again supports core pathway independent roles of Pk^Pk^ and Pk^Sple^ in orienting microtubules.

Notably, Pk^Sple^ cannot organize microtubules in *ft dachs* mutant wings [54]. This has led to a model in which Ft–Ds align microtubules along the proximodistal axis of the wing and anterior compartment of the abdomen, and Pk^Pk^ and Pk^Sple^ then control polarity of microtubule plus ends relative to Ft–Ds polarity. However, the mechanism by which the polarities are aligned remains to be elucidated. A role for the Pk^Pk^ isoform in orienting microtubules downstream of Ft–Ds is further suggested by experiments in which Ds is overexpressed in a gradient in the distal region of the pupal wing. Microtubule orientation and trichome polarity is reversed [73,121,128], and this reversal of trichomes is lost in a *pk^pk^* mutant but not in a *pk^sple^* mutant [121]. This might imply that the Pk^Pk^ isoform is necessary for the reversals in microtubule and trichome polarity caused by reversing the Ds gradient.

In summary, the evidence suggests that Pk isoforms influence the axis of microtubule orientation. In some cases this appears to be downstream of Ft–Ds, but how the two pathways are linked remains mysterious.

## 7. Polarization of cuticular structures by Ft–Ds independently of the core proteins

Experiments in the abdomen have shown that Ft and Ds can polarize hairs and bristles independently of the core proteins: for instance clones overexpressing Ft can repolarize wild-type cells lacking Fmi or Fz [107,117]. Thus while the core proteins may be the primary cue for hair orientation in this tissue, Ft–Ds can orient hairs in their absence. It was also reported that in adult wings lacking core protein activity, trichome swirling patterns correlate with asymmetric localization of Ds and these swirling patterns are altered if Ds activity is knocked down in pupal life [88]. Thus, Ft–Ds appear able to influence trichome polarity here as well.

The ability of Ft–Ds to organize the cytoskeleton independently of the core proteins has been confirmed by studies of the denticle belts on the embryonic and larval epidermis (figure 6*d*). These are segmented structures, where each segment has multiple rows of denticles, and each row has a distinct polarity. In the embryo, the denticles point posteriorly, apart from those in rows 1 and 4, that point anteriorly [134,135]. The core proteins localize asymmetrically to anterior–posterior cell edges, but loss of core protein activity has relatively little effect on denticle orientation [107,134–136]. However, in the embryo and larva, denticle polarity correlates with asymmetric localization of Ds and D [129,137,138], and loss or overexpression of Ft–Ds severely disrupts denticle belt polarity [107,136–139]. Fj is highly expressed in the tendon cells which are posterior to rows 1 and 4 in the larval epidermis, which may explain the pattern of denticle polarity [140]. Furthermore, overexpression of Ds can reorient denticle belts in the absence of Fz, indicating that it is not acting via the core pathway [136]. However, loss of both Ds and Fz has a stronger effect on denticle polarity than loss of Ds alone [107,136,138], suggesting some redundancy between the core and Ft–Ds pathways.

The mechanism by which Ft and Ds can organize denticle belts in the larva or hairs in the abdomen is unknown. Like trichomes and hairs, denticles are actin-containing cell protrusions that require the effector proteins In, Fy, Frtz and Mwh for correct polarity [22,134,135,141]. Whether asymmetric localization of Ft–Ds in the abdomen and denticle belts can direct asymmetric localization of the effector proteins in the absence of the core proteins remains to be determined.

One way in which Ft and Ds might influence the polarization of cuticular structures could be by regulating cell shape and alignment. In wild-type embryos, cells are elongated on the dorsoventral axis and microtubules are aligned on this long axis [129,138]. In the absence of Ft, cells fail to elongate on the dorsoventral axis, and microtubules are disorganized [138]. Ft–Ds also have a role in regulating cell alignment in the abdomen. Histoblast cells in the abdomen are initially disorganized, and they align uniformly over time, such that the long axis aligns along the mediolateral axis [77]. Multiple actin protrusions grow out of each cell, orthogonal to the axis of cell alignment. In *ds* mutants, cell alignment is disrupted, but trichomes still grow out from the abnormally oriented long axis of the cell [77]. An attractive model would be that trichomes or denticles preferentially form on the long edge of the cell, and the effector proteins would be necessary in situations where the ‘long edge rule’ needs to be over-ridden or reinforced. Nevertheless, regulating cell shape would only be sufficient to determine an axis of polarity, but not the overall direction (i.e. anterior or posterior). As Ft–Ds gradients control the direction of polarity there must also be a linkage (direct or indirect) between asymmetric localization of Ft–Ds and the site of actin protrusions.

Ft and Ds may also have effects on ommatidial orientation via the transcriptional co-repressor Atrophin (Atro). Atro binds to the intracellular domain of Ft, and loss-of-function *Atro* clones cause non-autonomous inversions of ommatidia on polar clone boundaries [95,142,143]. However, Atro regulates expression of components of multiple signalling pathways, as well as Ft and Fj [95,144–146], so it is unclear whether the effects of Atro on ommatidial orientation are direct or due to feedback regulation of Ft–Ds pathway activity.

## 8. Ft–Ds independent inputs into the core pathway

**Figure 8.**
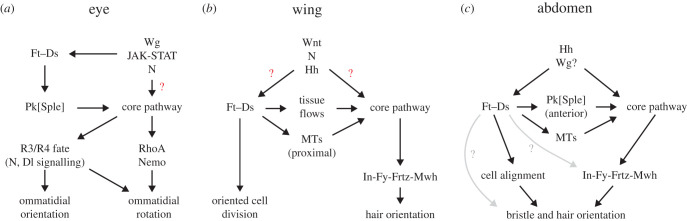
Inputs into the Ft–Ds and core pathways in the *Drosophila* eye, wing and abdomen. (*a*) In the eye, morphogen signalling regulates Ft–Ds pathway expression. Ft–Ds promote asymmetric localization of the core proteins via Pk^Sple^, but there are likely to be unidentified independent inputs into the core pathway (red question mark). Asymmetric localization of the core proteins regulates Notch signalling in the R3 and R4 photoreceptors and ommatidial rotation via RhoA and Nemo. (*b*) In the wing, multiple morphogen signalling pathways are thought to have independent inputs into the Ft–Ds and core pathways (red question marks). Ft–Ds regulate core protein localization indirectly, by regulating tissue flows and cell packing and by regulating microtubule alignment in the proximal region. The core pathway acts through effector proteins In, Fy, Frtz and Mwh to regulate the localization of trichomes, while Ft and Ds regulate oriented cell division via Dachs. (*c*) In the abdomen, Hh signalling has independent inputs into the Ft–Ds and core pathways. In the anterior compartment, Ft–Ds promote asymmetric localization of the core proteins via Pk^Sple^, and by regulating microtubule alignment. The Ft–Ds pathway and the core pathway can independently regulate hair and bristle orientation.

In the eye, genetic evidence suggests that Ft–Ds can act upstream of the core proteins via Pk^Sple^. Nevertheless, in this tissue there are also likely to be Ft–Ds independent inputs into the core pathway. Suppressing Hippo signalling via a reduction in Yki activity suppresses the overgrowth seen in *ft* or *ds* mutant eyes, and this also partially suppresses the planar polarity defects [76]. This implies that the core proteins are capable of responding to other cues present in this tissue, and that these cues are disrupted by tissue overgrowth. The identity of these cues is unknown, but multiple signalling pathways have graded activity in the eye disc. There is a dorsoventral gradient of Wingless (Wg) that is high at the poles, and loss of Wg signal transduction in clones gives polarity inversions on the equatorial side of the clone [147,148]. There is also a dorsoventral gradient of JAK/STAT activity, where the ligand Unpaired (Upd) is high at the equator, and loss of JAK/STAT signalling in clones gives polar polarity inversions [149]. Finally, Notch (N) activity is high at the equator [150–152]. Fj expression is regulated by Wg, Upd and N [149,152]; and Ds expression by Wg [92], but one or more of these signalling pathways could also have independent inputs into the core proteins (figure 8*a*).

In most of the wing, there appears to be no direct coupling of the core proteins to Ft–Ds; but as discussed above Ft–Ds appear to affect the core indirectly by affecting tissue flows and growth, and in the proximal wing they may direct core protein localization by regulating microtubule alignment. The identity of other cues to the core proteins is not clear. Fz proteins are receptors for Wnt ligands [153], and a number of Wnt proteins are expressed at high levels at the dorsoventral boundary (the future wing margin) [154–158]. However, recent experiments argue against a role for diffusible Wnts in affecting core protein asymmetry [114,157–159]. Disruption of Notch at the dorsoventral boundary and Hedgehog (Hh) at the anteroposterior boundary also have mild effects on core protein asymmetry [114]. Genetic evidence also suggests coupling of the core pathway to upstream cues occurs early in development [66,67,160]. It was therefore proposed that a combination of cues may feed into the core pathway and that the pattern of asymmetry is maintained during growth and then altered and refined by mechanical forces and tissue flows (figure 8*b*) [114,115].

As in the wing, hair orientation defects in the abdomen in *ft* or *ds* mutants can be partially suppressed by loss of Dachs activity [78,119], or overexpression of Ft lacking the extracellular domain [109,110]. This suggests that, as in the eye and wing, there are Ft–Ds independent inputs into the core proteins. In the abdomen, Hh is expressed throughout the posterior compartment and in inverted gradients in the anterior compartment [161,162]. This induces Wg gradients in both anterior and posterior compartments [163]. Interestingly, clones in which Hh signalling is constitutively activated can repolarize hairs in the absence of either Ds or Fz, suggesting independent inputs of Hh into Ft–Ds and the core pathway (figure 8*c*) [107].

## 9. Ft–Ds and core pathway interactions in other animal models

In other animal models, as in flies, the Ft–Ds and core pathways regulate common planar polarized processes. For example, mouse mutants in both pathways affect hair cell orientation in the inner ear, cochlear extension, and cause kidney cysts and various skeletal defects (reviewed in [1,164,165]). Is there evidence for coupling between the two pathways? Vertebrate Pk homologues are similar to the Pk^Pk^ isoform in flies and therefore lack the conserved N-terminus of the Pk^Sple^ isoform that binds to fly Ds and Dachs, and there is also no known Dachs homologue in vertebrates. This would appear to rule out a direct linkage between the two pathways as seen in some *Drosophila* tissues.

The possibility of indirect linkages between the Ft–Ds and core pathways has been poorly explored. Indeed, in some non-fly models there is clear evidence that they act independently on the same processes. One example is facial branchiomotor (FBM) neuron migration, where neurons migrate caudally from rhombomere 4 along the midline and then laterally in rhombomere 6. The core pathway is needed for caudal migration (reviewed in [164,166]), while the Ft–Ds homologues are needed for lateral migration [167]. Similarly, in planaria, the core pathway polarizes on the anteroposterior axis and Ft–Ds on the mediolateral axis to orient ciliary rootlets on the epidermis [168]. In the mouse kidney, loss of Fat4 does not affect core protein localization, and mutations in the Ft–Ds and core pathways have synergistic effects [169], again suggestive of parallel rather than sequential action.

As in flies, there is also emerging evidence from a number of other systems that Ft–Ds regulate cell shape, cell orientation and microtubule dynamics. The mouse homologues Fat4 and Dchs1 regulate oriented cell division in kidney tubules [169–172]. They also regulate cell orientation in the condensing mesenchyme in the developing mouse sternum [170,173], and in the mesenchymal cells that cluster below the emerging villi in the gut [174]. Cell orientation in lymphatic valve morphogenesis is also defective in Ft4 and Dchs1 mutants [175,176], although here the core pathway also regulates cell rearrangements [177,178]. Furthermore, cell shape and alignment of epithelial cells in the body column of the cnidarian *Hydra* is regulated by HyFat and HyDs [179]. Finally, in zebrafish Dchs1b regulates microtubule turnover via AuroraB [180,181].

Taken together, there is little evidence thus far from non-fly models that the core pathway is regulated by the Ft–Ds pathway. A few studies support the hypothesis that the core pathway and Ft–Ds largely act independently. However, possible linkages between the pathways have not been studied in depth and so this will merit further investigation.

## 10. Conclusion and future directions

To summarize, the existing literature suggests that there are both direct and indirect links between the Ft–Ds system and the core proteins in flies. In tissues where Pk^Sple^ is present—the eye, anterior abdomen and during posterior wing ridge specification—this appears to be a strong cue that couples Ft–Ds directly to the core proteins. In the absence of Pk^Sple^ the two pathways appear largely independent, but Ft–Ds appear to regulate core protein asymmetry indirectly by affecting microtubule orientation and tissue organization. Studies of how Ft–Ds regulate cell behaviour and microtubule orientation will, therefore, be of much interest. Furthermore, how Pk isoforms regulate microtubule orientation and how this is linked to Ft–Ds activity is an area that requires further study.

It has become clear that the core pathway has inputs independent of Ft–Ds in all studied tissues. Understanding these cues will be of great importance, but unravelling the contribution of multiple signalling pathways that also cross-regulate each other will be challenging. This could be addressed by examining the effects of acute manipulation of different pathway activities on core protein localization and stability. Finally, in many tissues both Ft–Ds and the core pathway can influence polarization of particular cellular structures: the contribution of each pathway appears to vary, with one pathway usually dominating. Understanding how different pathways feed into the same downstream events will be an exciting area of future study.

## Data accessibility

This article does not contain any additional data.
